# Signaling Functions of Intramembrane Aspartyl-Proteases

**DOI:** 10.3389/fcvm.2020.591787

**Published:** 2020-12-14

**Authors:** Alkmini A. Papadopoulou, Regina Fluhrer

**Affiliations:** ^1^Biochemistry and Molecular Biology, Institute of Theoretical Medicine, Medical Faculty, University of Augsburg, Augsburg, Germany; ^2^German Center for Neurodegenerative Diseases (DZNE), Munich, Germany

**Keywords:** intramembrane proteolysis, signal peptide peptidase, signal peptide peptidase-like, presenilin, cellular signaling, GxGD aspartyl proteases

## Abstract

Intramembrane proteolysis is more than a mechanism to “clean” the membranes from proteins no longer needed. By non-reversibly modifying transmembrane proteins, intramembrane cleaving proteases hold key roles in multiple signaling pathways and often distinguish physiological from pathological conditions. Signal peptide peptidase (SPP) and signal peptide peptidase-like proteases (SPPLs) recently have been associated with multiple functions in the field of signal transduction. SPP/SPPLs together with presenilins (PSs) are the only two families of intramembrane cleaving aspartyl proteases known in mammals. PS1 or PS2 comprise the catalytic center of the γ-secretase complex, which is well-studied in the context of Alzheimer's disease. The mammalian SPP/SPPL family of intramembrane cleaving proteases consists of five members: SPP and its homologous proteins SPPL2a, SPPL2b, SPPL2c, and SPPL3. Although these proteases were discovered due to their homology to PSs, it became evident in the past two decades that no physiological functions are shared between these two families. Based on studies in cell culture models various substrates of SPP/SPPL proteases have been identified in the past years and recently-developed mouse lines lacking individual members of this protease family, will help to further clarify the physiological functions of these proteases. In this review we concentrate on signaling roles of mammalian intramembrane cleaving aspartyl proteases. In particular, we will highlight the signaling roles of PS via its substrates NOTCH, VEGF, and others, mainly focusing on its involvement in vasculature. Delineating also signaling pathways that are affected and/or controlled by SPP/SPPL proteases. From SPP's participation in tumor progression and survival, to SPPL3's regulation of protein glycosylation and SPPL2c's control over cellular calcium stores, various crossovers between proteolytic activity of intramembrane proteases and cell signaling will be described.

## Introduction

Proteolysis is a non-reversible post-translational protein modification and can regulate the function of a protein by contributing to either its maturation and activation, or its degradation. Consequently, the enzymes responsible for proteolysis, the proteases, play a crucial role in regulating key cellular processes, including molecular signaling both within the cell and from cell-to-cell.

Intramembrane proteolysis describes proteolytic processing within the transmembrane domain of membrane-spanning proteins (1). Despite that the process of proteolysis was discovered already in the nineteenth century (2, 3), it had been considered possible only within a fully aquatic environment for the great majority of the past. Only within the past 20 years intramembrane proteolysis became a well-accepted concept and since then the number of proteins undergoing intramembrane proteolysis has increased exponentially (1, 4–7).

Intramembrane cleaving proteases (I-CLiPs) are all multipass transmembrane proteins that cleave their substrates within or very close to the membrane bilayer by most-likely creating a cavity, where water can approach the peptide bond allowing hydrolysis (8–11). I-CLiPs are categorized based on their catalytic centers. The four classes of I-CLiPs are: aspartyl proteases, metalloproteases, serine proteases and glutamyl proteases (12, 13).

This review focuses on mammalian intramembrane aspartyl proteases that, according to a conserved active site motive, are termed GxGD aspartyl proteases (14). This protease class comprises two families, the presenilins (PSs) and the signal peptide peptidases (SPPs). The first family has two members, PS1 and PS2, while the second family has five members: signal peptide peptidase (SPP) and its homologous enzymes, the SPP-like (SPPL) proteases SPPL2a, SPPL2b, SPPL2c, and SPPL3 (15–17). Since the cleavage mechanism and substrate selection of these proteases have been covered in great detail lately (6, 18, 19), this article will concentrate on recent findings that provide insight in how GxGD proteases affect intra- and intercellular signaling. To this end, we specifically summarize cleavage and function of specific validated substrates by aspartyl intramembrane proteases.

## Shedding and Intramembrane Proteolysis

**Figure 1:**
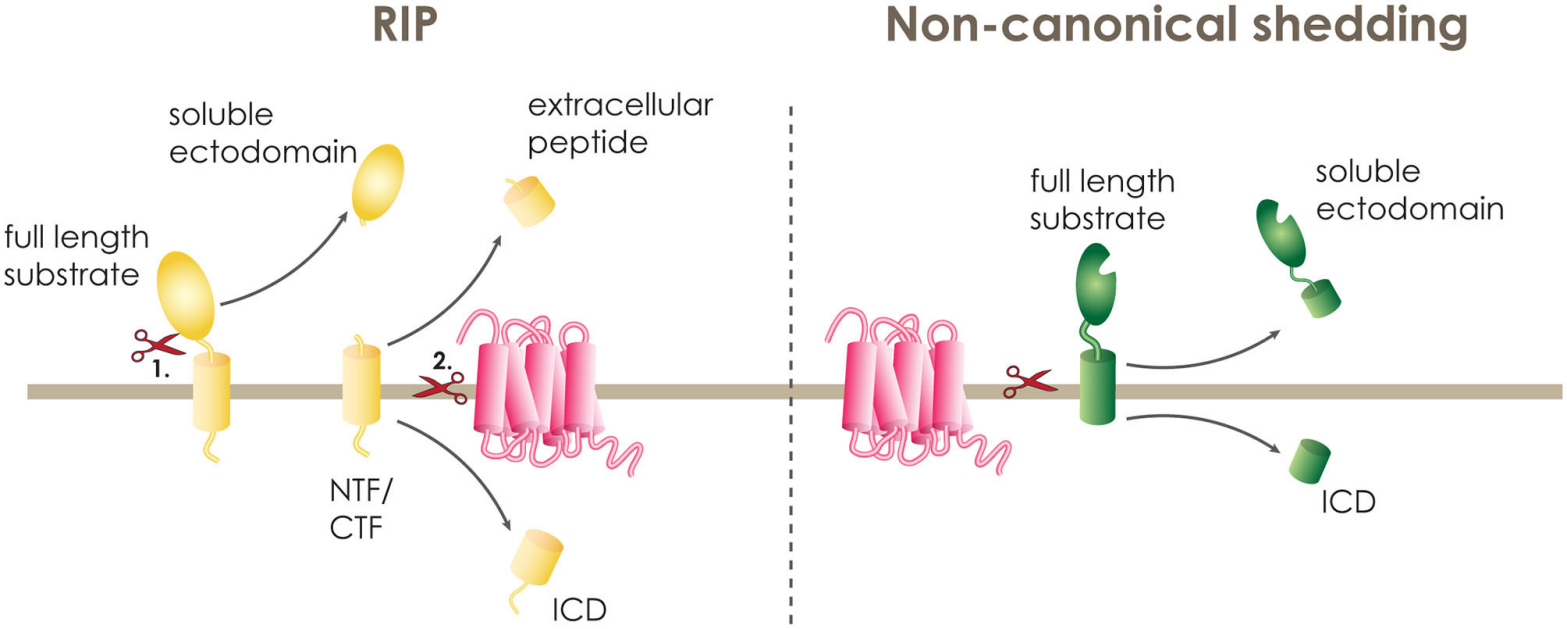
Intramembrane proteolysis. A transmembrane protein can be cleaved within its transmembrane domain either in a two-step procedure termed regulated intramembrane proteolysis (RIP) or in one step by non-canonical shedding. During RIP, the full length substrate (yellow) is cleaved first (1.) by a sheddase releasing the soluble ectodomain and leaving the N-terminal or C-terminal fragment (NTF or CTF) on the membrane. The NTF or CTF is then (2.) cleaved by the intramembrane cleaving protease (pink), releasing the extracellular peptide and the intracellular domain (ICD). At non-canonical shedding, the full length substrate (green) is directly cleaved within or at the border of the transmembrane domain releasing the soluble ectodomain and the ICD.

A large number of human single-pass transmembrane proteins undergo proteolytic removal of their extracellular domain (ECD), a process termed ectodomain shedding, before they become substrates of intramembrane proteases. This ectodomain shedding is performed by membrane bound and soluble proteases of the extracellular matrix, termed canonical sheddases. If the cleaved ECD is secreted from the cell as a soluble fragment, these proteases are frequently also called secretases. Some of the most well-studied canonical sheddases that cleave their substrates within the luminal juxtamembrane domain include the β-site APP cleaving enzyme 1 and 2 (BACE 1 and 2) and a disintegrin and metalloproteinase (ADAM) family (20). Following the release of the soluble ectodomain, shedded substrates comprise a C- or N-terminal membrane-spanning fragment (CTF or NTF) that is significantly shortened and can only then undergo intramembrane proteolysis (Figure 1). This two-step proteolytic cascade is termed regulated intramembrane proteolysis (RIP) and was first described in 2000 (21).

PS1 and SPPL2b have a strong preference of cleaving substrates with short ectodomains that do not exceed 60 amino acids in length. In particular, some of the known PS1 substrates, and also tail anchored proteins, which are substrate to SPP and SPPL2c mediated intramembrane cleavage (22–25), have a naturally short extracellular domain, and, thus, can be cleaved directly by the I-CLiP (19). Additionally, some I-CLiPs are known to directly accept substrates with long and bulky ECDs. Most of these proteases belong to the family of serine intramembrane proteases called rhomboids (26), but also SPPL3 is performing direct cleavage of substrates with long ectodomains (Figure 1) (18, 27–29). These I-CLiPs are referred to as “non-canonical” sheddases (20).

In addition, multipass TM proteins can also undergo RIP. For a single pass transmembrane protein, the cleavage within or at the border of the transmembrane domain leads to the release of an extracellular fragment at the luminal or extracellular side, and an intracellular domain (ICD). While, for multipass transmembrane proteins, the first cut leads to the breakage of a loop and the second cut takes place within the transmembrane domain (27).

## GxGD Aspartyl-Proteases

PS1 and 2 were the first GxGD aspartyl-proteases discovered. Their discovery followed the research into the pathogenesis of Alzheimer's disease, the most prevalent form of dementia (30–34).

The second family of GxGD aspartyl-proteases, the SPP/SPPLs, were discovered by database sequence homology analysis based on the PS sequence, as well as by biochemical methods (15–17). All seven human GxGD aspartyl proteases have a multi-transmembrane domain protein structure consisting of nine transmembrane domains (18). The two aspartic acids that comprise their active site, are located within conserved transmembrane domain motifs. The first aspartic acid is located in a YD motif in transmembrane domain 6. The second aspartic acid is part of the GxGD motif, found in transmembrane domain 7, which also gives the name to this class of proteases. These aspartic acids are essential for the catalytic activity of the proteases, as mutating either of the aspartic residues almost completely abolishes catalytic activity of the protease (17, 28, 35–39).

Another characteristic that is shared by all GxGD aspartyl proteases, is a conserved PAL motif in transmembrane domain 9. Mutations in the PAL motif have been shown to negatively affect the proteolytic ability of at least SPP and PS, however its purpose has so far only been investigated in the γ-secretase complex (18, 40–42). The involvement of the PAL motif in the architecture of the active site and substrate recognition had been speculated for many years (43). Recent analysis of the 3D structure of γ-secretase cross-linked with its substrate APP confirmed that the PAL motif is involved in substrate recognition and even showed that the PAL motif is directly binding a β-strand of the substrate (44). It is, thus, tempting to speculate that the PAL motif functions as a gate keeper for the substrate to enter the active site and although it has not been investigated so far, it could serve a similar purpose in SPPLs.

A key difference between the PS and the SPP/SPPL family, is their reversed membrane topology. The N-termini of PSs are located in the cytosol, while SPP/SPPLs have their N-termini in the lumen or extracellular space (45, 46). As this leads to inverted topology of their active sites, it might account for their specificity of cleaving substrates with opposite membrane orientation. PSs only cleave type-I transmembrane proteins, with their N-termini in the lumen or extracellular space, while SPP/SPPLs only cleave type-II transmembrane proteins, with their N-termini in the cytosol (6, 18).

Although some of the SPP/SPPLs are thought to undergo multimerization and have been observed as homodimers or tetramers, they most likely do not require additional cofactors for their catalytic activity (46–48). On the other hand, PSs are only active as part of the γ-secretase complex and favor endoproteolysis in their cytosolic loop between transmembrane domain 6 and 7, forming PS CTF and NTF to be active (19, 49).

**Figure 2:**
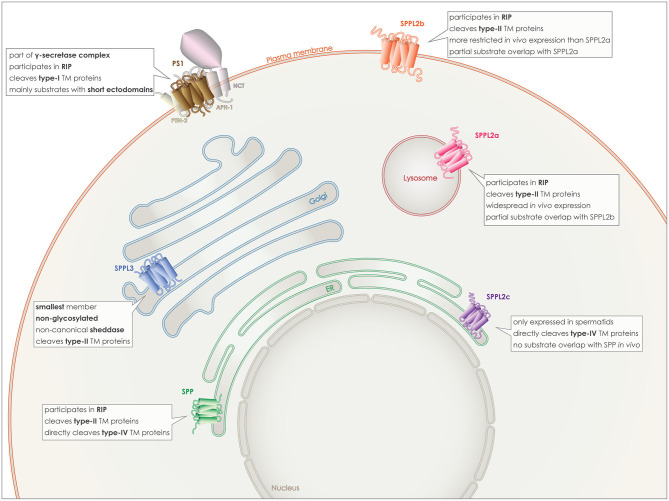
Localization and characteristics of GxGD aspartyl proteases. PS1 (brown), localizes to the plasma membrane, as part of the γ-secretase complex with APH-1, PEN2, and NCT. SPP (green) localizes to the ER membrane, as SPPL2c (purple), however despite the *in vitro* partial overlap of substrates, they do not share similar substrate spectra *in vivo* and SPPL2c has a very limited expression pattern *in vivo*. SPPL2a (red) localizes to the lysosomal/late endosomal membrane and although *in vitro* it shares some substrates with SPPL2b (orange), the latter localizes to the plasma membrane. Finally, SPPL3 (blue) is the smallest member and localizes to the Golgi membrane.

The γ-secretase complex is formed by PS1 or 2 together with three additional proteins, presenilin enhancer 2 (PEN-2), anterior pharynx defective-1 (APH-1), and nicastrin (NCT) (Figure 2). PEN-2 is a two-transmembrane domain protein that is necessary both for endoproteolysis of PSs and for stabilization of PS CTF and NTF (50–52). APH-1 comprises seven transmembrane domains and a GxxxG motif is believed to act as a connecting subunit holding together NCT with PS NTF and CTF (53). The last member of the complex, NCT, is a single pass transmembrane protein with a large and heavily glycosylated ectodomain. Due to the size, position and charge of the NCT's ectodomain, it is proposed to act as the “gate keeper” in the complex, recognizing the substrates and allowing only proteins with a short ectodomain to enter the active site (54, 55).

Another interesting characteristic of the γ-secretase complex is its unusually slow proteolysis speed. This characteristic was initially observed on the processing of the β-amyloid precursor protein (APP) (56) and was later confirmed on the processing of the epithelial cell adhesion molecule (EpCAM)-CTF (57). It is not clear if the low speed is due to substrate recognition, binding or the proteolysis itself, however similar results had been obtained previously for rhomboid proteases (58). It has not yet been investigated whether this applies to the other members of the GxGD class of proteases but it does not appear as if these proteases have been purposed for a fast turnover rate.

PS 1 and 2 homologs are roughly 65% identical and both can be part of γ-secretase complexes, cleaving the same substrates *in vitro* (59). Familial mutations causing Alzheimer's disease have mainly been detected in *PSEN1*, the coding gene for PS1 and to a lesser degree in *PSEN2* (30–34). The γ-secretase complex containing PS1 has been reported to have a higher affinity for APP, and also to localize to different subcellular compartments than PS2 containing complexes. PS1 complexes can be detected on the plasma membrane (Figure 2), while PS2 complexes are targeted to late endosomes and lysosomes (60, 61).

The key product of γ-secretase cleavage that led to its discovery is the amyloid β-peptide (Aβ). Aggregation of Aβ molecules in the central nervous system is a hallmark of Alzheimer's disease (14, 62–64). Production of Aβ is a well-studied process that results from the amyloidogenic processing of the type-I membrane protein APP, however it is not the only possible product of the γ-secretase processing (27). During RIP, APP is initially shedded by either ADAM10 or BACE1 (20). Each sheddase prefers a distinct cleavage site and cleavage by ADAM10 (α-secretase) (65) releases a longer soluble APP (sAPPα) and a shortened CTFα. This is considered the non-amyloidogenic processing as cleavage of CTFα by γ-secretase releases APP ICD (AICD) and the p3 peptide, which is smaller, less hydrophobic and less prone to aggregation than Aβ (66). On the contrary, shedding of APP by BACE1 (β-secretase) releases a shorter sAPPβ and a longer CTFβ. Processing of CTFβ by γ-secretase results in release of the same AICD and the Aβ peptide, which is longer than p3 and prone to aggregation making this the amyloidogenic processing (10, 67–69). Although AICD has been suggested to possess transcriptional functions as seen in other ICDs (70, 71), its fast degradation stands against those hypotheses.

The SPP/SPPL family members are characterized by more heterogeneity and less substrate overlapping than the PSs. SPP, the first discovered protease of this family, is retained to the endoplasmic reticulum (ER) by a C-terminal KKXX-sequence (17). From the three SPPL2 proteins, SPPL2a and SPPL2b share more similarities and some substrate overlap, especially *in vitro*. SPPL2a is mostly transported to the lysosomes/late endosomes via its C-terminal YXXø sorting signal, while SPPL2b most likely localizes to the plasma membrane (Figure 2) (38, 72). Additionally, their expression patterns within the organism differ with SPPL2a being expressed more ubiquitously and SPPL2b mainly in the central nervous system, bone marrow and lymphoid system (73).

SPPL2c has a different localization in the cell, as well as in the whole organism and also differs in its substrate spectrum from the other two SPPL2 proteases. For a long time, it was considered to be a pseudogene and a pseudoprotease, as no SPPL2c protein levels were detected *in vivo* and no substrates had been identified. This concept changed last year when two papers demonstrated SPPL2c protein expression and identified physiological substrates of this protease. Expression of the protein *in vivo* was found only in a specific cell type within the testis, while its cellular localization is in the ER/ER-Golgi intermediate compartment (ERGIC) (Figure 2) (24, 25).

Last but not least, SPPL3 is the smallest member of this family, it is localized in the Golgi and it is not glycosylated (Figure 2). In contrast to all other GxGD aspartyl proteases, SPPL3 acts exclusively as non-canonical sheddase and directly cleaves full-length substrates with very long ectodomains, resulting in the release of soluble ectodomains to the Golgi lumen and, consequently, also to the extracellular space (28, 29, 74).

## Intramembrane Proteolysis and Signaling

Intramembrane proteolysis is capable of directly controlling the abundance of certain membrane proteins, and thus, also their local activity. In this scenario, reduced proteolytic cleavage would result in increased presence of the substrate on the membrane and vice versa. The accumulating substrate of intramembrane proteolysis could be either the full length protein, in the case of non-canonical shedding, or the membrane bound remaining product of shedding, in the case of RIP. If this substrate's role positively impacts on signaling while spanning the membrane, then reduction of the protease activity, would result in increased signaling. Such is the case, for example, with signaling induced by the accumulation of Lectin-like oxidized LDL receptor 1 (LOX1) NTF when the activity of SPPL2a or SPPL2b is reduced (75). On the contrary, if this substrate has an inhibitory function by its presence on the membrane, as for example does FKBP8, a substrate of SPP, increased proteolysis would decrease its inhibitory function and boost signaling (76).

However, not all substrates fulfill their functions while attached to the membrane. In certain cases, cleavage of the substrate plays an integral part in the signaling cascade and is needed for a signal to be passed forward via the cleavage products. In these cases the various products of cleavage can function as distinct signaling molecules. In such scenarios reducing proteolytic cleavage would actually inhibit the signal transmission. For instance, when a transmembrane substrate undergoes cleavage in the secretory pathway or at the cell surface, a part of it can be released to the compartment lumen or extracellular space. In particular protein domains released to the extracellular space may serve a purpose by binding to a receptor on a neighboring or even far distant cell in the body. This scenario is most common for canonical sheddases, such as the shedding of tumor necrosis factor-α (TNFα) by ADAM17 (77, 78), but is also a function of non-canonical sheddases, as seen for shedding of epidermal growth factor (EGF) by rhomboid-related protein 2 (RHBDL2). Although SPPL3 is also a non-canonical sheddase, no purpose has so far been identified for the secreted extracellular domains of its substrates (28, 29).

At the same time, intramembrane cleavage also triggers the release of the substrate's intracellular domain. Although these intracellular peptides are often unstable and undergo rapid degradation, in certain cases they are known to translocate to the nucleus where they can either directly or in collaboration with other transcription factors activate or deactivate the transcription of target genes. A well-known example in this category is the release of the Notch ICD by γ-secretase, which translocates to the nucleus and induces the expression of specific genes (79).

Intramembrane proteases can have also an indirect effect on signaling. Although there are numerous such possibilities, some key indirect effects of I-CLiPs on signaling include changes in localization, trafficking and glycosylation of signaling molecules, as well as influences on calcium signaling. I-CLiPs are capable of affecting the abundance of key trafficking molecules by, for example, cleaving key trafficking factors, such as the soluble N-ethylmaleimide-sensitive factor (NSF) attachment protein receptor proteins (SNAREs), which are substrates of both SPP and SPPL2c (25, 80). In that case, increased I-CLiP activity would result in decreased trafficking of various cargo molecules and, thus, certain signaling molecules cannot reach their target localization either within or outside the cell. Glycosylation of secretory proteins can be affected by, for example, proteolytically inactivating glycosylating enzymes in the cell, as is the case with SPPL3 dependent cleavage of glycosidases and glycosyltransferases (28, 29). Thus, changes of the protease activity can directly impact on the glycosylation pattern of a cell.

Beside the knowledge of the general impact of intramembrane cleavage on signaling, some specific cellular processes that are influenced by GxGD aspartyl proteases have been in focus of research in the past years.

## Intramembrane Proteolysis in Signaling of the Vascular System

Very recently involvement of SPPL2a/b in the signaling of the vascular system was discovered (75). In this study, the authors demonstrate that LOX1 is initially cleaved by either ADAM10 or lysosomal proteases, and the remaining membrane associated LOX1 NTFs are cleaved by SPPL2a/b. Cleavage of LOX1 NTFs by SPPL2a/b takes place either on the cell surface or in the lysosomes and, consequently, leads to the reduction of LOX1 NTF levels and the release of LOX1 ICD into the cytosol. Although the LOX1 ICD function remains enigmatic, accumulation of LOX1 NTF induces proatherogenic and profibrotic signaling and therefore their removal is atheroprotective. For more details regarding the participation of SPPL2a/b in the signaling process of LOX1 and their atheroprotective function, please refer to a detailed review by Mentrup et al. included in this issue.

The invariant chain (CD74) of the major histocompatibility class II complex (MHCII), another substrate of SPPL2a, appears to be connected to the formation of atherosclerosis, CD74 has been validated to undergo RIP *in vivo* being initially shedded by serine or cysteine proteases, releasing the CD74 soluble ectodomain (CD74 ECD). SPPL2a then cleaves the remaining NTF intramembranously releasing the CD74 ICD (6, 81). The main purpose of CD74 is related to the function of the immune system, however levels of CD74 were found to be increased in human atherosclerotic plaques contributing to the pathology (82). Another study using low-density lipoprotein receptor-deficient mice (Ldlr^−/−^) as a model of familial hypocholesterolaemia, showed that additional deficiency of CD74 (Ldlr^−/−^ Cd74^−/−^) had a protective effect on atherosclerosis (83). Although it was shown that this effect most likely was connected to the impairment of the adaptive immune system, it has so far not been investigated which part of the CD74 protein is responsible for this effect. It remains to be seen whether lack of the full length CD74 is necessary, or targeting the release of one of the products of RIP (CD74 ECD, NTF, or ICD) would be sufficient for the protective role. The soluble CD74 ECD has already been connected to signaling functions in context of the macrophage migration inhibitory factor (MIF) related to cardiovascular diseases (84). The function of the CD74 intramembrane cleavage products, however, so far, remains enigmatic.

**Figure 3:**
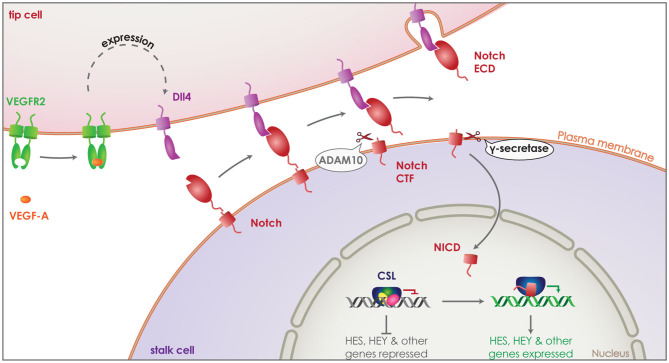
Role of γ-secretase in Notch signaling. Vascular endothelial growth factor A (VEGF-A) (orange) activates endothelial cells transforming them to tip cells during angiogenesis. Binding of VEGF-A to VEGF receptor 2 (VEGFR2) (green) induces expression of delta-like-4 (Dll4) (purple). Dll4 acts as a signaling molecule, binding to the Notch receptor (red) on neighboring endothelial cells. This activates the Notch receptor and induces its cleavage by ADAM10, releasing the ectodomain that gets internalized attached on Dll4. The remaining Notch CTF is cleaved by γ-secretase, releasing the Notch ICD (NICD) into the cytosol of the endothelial cell. NICD translocates to the nucleus and binds transcription factor CSL (blue), switching it from a repressor to an activator and thus, inducing the expression of HES and HEY family genes. This suppresses activation of the Notch expressing endothelial cell, turning it to a stalk cell and defining the new vessel.

The formation of the vascular system is crucial both during development and regeneration, while it can be detrimental under pathological conditions, such as cancer (85, 86). New blood vessels are created via endothelial cell sprouting and proliferation, a process that needs to be tightly regulated via cell-to-cell communication (87, 88). Endothelial cells are activated by angiogenic signals, primarily through vascular endothelial growth factor A (VEGF-A), becoming the tip cells of angiogenesis. This initiates the expression of a series of proteins including VEGF receptors 2 (VEGFR2) and delta-like-4 (Dll4) (Figure 3). Some of these proteins act as signaling molecules on neighboring endothelial cells suppressing their activation and turning them to stalk cells, defining the new vessel. The main mechanism by which this is achieved is the activation of the NOTCH receptors on stalk cells by their ligands, such as Dll, which in turn can have an effect on the expression of VEGFR on neighboring tip cells (89).

PSs, as part of the γ-secretase complex, are connected to the vascular system and angiogenesis through a number of their substrates that include the receptors Notch, VEGFR-1 and others (5). In addition, non-proteolytic functions of presenilin related to its phosphorylation or the translocation of these receptors have also been discussed in the context of vasculature and angiogenesis but will not be covered further here (90–92).

The Notch signaling pathway is highly conserved in evolution and it has been studied in great depths. It has a clear connection to both the physiological formation of the vascular system, promoting the formation of arteries (93), but also to pathological angiogenesis, as seen in breast cancer and other types of cancer (94–96). The importance of the NOTCH pathway in the health of the vascular system can be confirmed as defects in this pathway can cause Cerebral Autosomal Dominant Arteriopathy with Subcortical Infarcts and Leukoencephalopathy (CADASIL) (91).

After activation of one of the four Notch receptors by a neighboring cell presenting one of Notch's transmembrane ligands, Dll 1, 3 or 4, Notch is cleaved first by ADAM10 and then by γ-secretase, releasing the Notch ICD (NICD) into the cytosol. NICD can translocate to the nucleus and bind the transcription factor CSL acting then as a transcriptional activation complex leading to the expression of downstream HES and HEY family genes (Figure 3) (79). It has been shown that Dll4 is the principle Notch ligand expressed by vascular epithelial cells and its haploinsufficiency is enough to cause a lethal phenotype in mice (97–99). Interestingly, inhibition of Notch CTF cleavage by a γ-secretase inhibitor is enough to reproduce an effect in Notch signaling similar to Dll4 haploinsufficiency (100). These results clearly demonstrate that γ-secretase cleavage is performed following the activation and shedding of the Notch receptor and is indispensable for the Notch ICD dependent signaling. In line with this, deletion of PS1 in mice (PS1^−/−^ mice) is sufficient to reproduce the embryonically lethal phenotype observed in Notch^−/−^ mice (101–103). While PS2^−/−^ mice are viable with a minor motor phenotype (104), PS1^−/−^ suffer embryonic death with severe axial skeleton (102) and central nervous systems defects (101), as observed in Notch^−/−^ mice (105, 106).

Interestingly, Notch ligands, including Dll and Jagged (Jag), have also been suggested to undergo RIP, with ADAM17 performing the first cut and γ-secretase releasing their ICDs (107). Although it is very stimulating that Notch and its ligands can undergo cleavage by the same machinery, the purpose of this mechanistic overlap or the fate of the released ligand ICDs remains unclear.

VEGFR1 is highly similar to VEGFR2 and has a very high affinity for VEGF, however its kinase activity is significantly reduced and plays a more variable role as it can regulate the VEGFR2 induced angiogenesis (108, 109). Pigment epithelium-derived factor (PEDF) is the most potent endogenous negative regulator of blood vessel growth and its ability to block the formation of new vessels seems dependent to VEGFR1 expression (90). Interestingly, it has been reported that VEGFR1 is not only cleaved by γ-secretase as part of RIP, but it also appears that this cleavage can be boosted by PEDF (90). Increased activity of γ-secretase in response to PEDF treatment could help VEGFR1 fulfill its purpose as negative regulator of VEGFR2-induced angiogenesis by participating in the trafficking and intracellular translocation of VEGFR1 CTF and inhibiting the phosphorylation of VEGFR1 following VEGF binding (92). Although γ-secretase has an effect on VEGFR1 function and leads to the production of a VEGFR1 ICD from the VEGFR1 CTF that is then degraded by the proteasome, the purpose and extent of this regulation, as well as whether γ-secretase has indeed a role in trafficking and phosphorylation is so far not clear under physiological conditions (92).

As PSs are involved in vasculature signaling and pathological angiogenesis is essential in the formation and progression of tumors, it is of great interest to understand how PSs and SPP/SPPLs may affect cancer progression.

## Intramembrane Proteolysis in Cancer-Associated Signaling

Pathological angiogenesis is needed for tumors to grow, as they require a high supply of nutrients and oxygen. The Notch receptor plays a key role for the formation of new vessels and it has been shown since many years that it is also an important contributor during malignant angiogenesis in both hematopoietic and solid tumors (110). Ligand-induced Notch signaling is often increased and dysregulated during pathological neoangiogenesis and targeting this signaling pathway can be achieved by targeting either the receptor itself (pan-Notch inhibitors and specific Notch receptor antibodies) or targeting ligands that are specific to the angiogenetic functions. As the most common ligands of Notch during angiogenesis that are also found to be upregulated in cancer, Dll4 and Jag1 have both been targeted for treatment of breast cancer tumors, with Dll4 being a more established focus point (111, 112).

It has been shown that ligand-induced signaling via the Notch receptor requires cleavage by γ-secretase for the production of NICD that then translocates to the nucleus and affects transcription (Figure 3) (79). Consequently, γ-secretase inhibitors are being evaluated for their ability to block this signaling pathway showing positive results in preventing tumor promotion and inducing tumor cell death both *in vitro* and *in vivo* (113–117). However, Notch inhibition via γ-secretase mainly works with additional treatments as it can only be tolerated for short times. This is partly due to the numerous substrates cleaved by γ-secretase, leading to many side-effects after prolonged inhibition (118, 119). Another important reason for balancing the intensity and extent of γ-secretase inhibition are the tumor-suppressing roles of Notch, due to its participation in definitive haematopoiesis (120) and T-cell development (121, 122). It has already been shown in mouse models that γ-secretase inactivation by NCT deletion can cause chronic myelomonocytic leukemia in a Notch-dependent manner (123).

**Figure 4:**
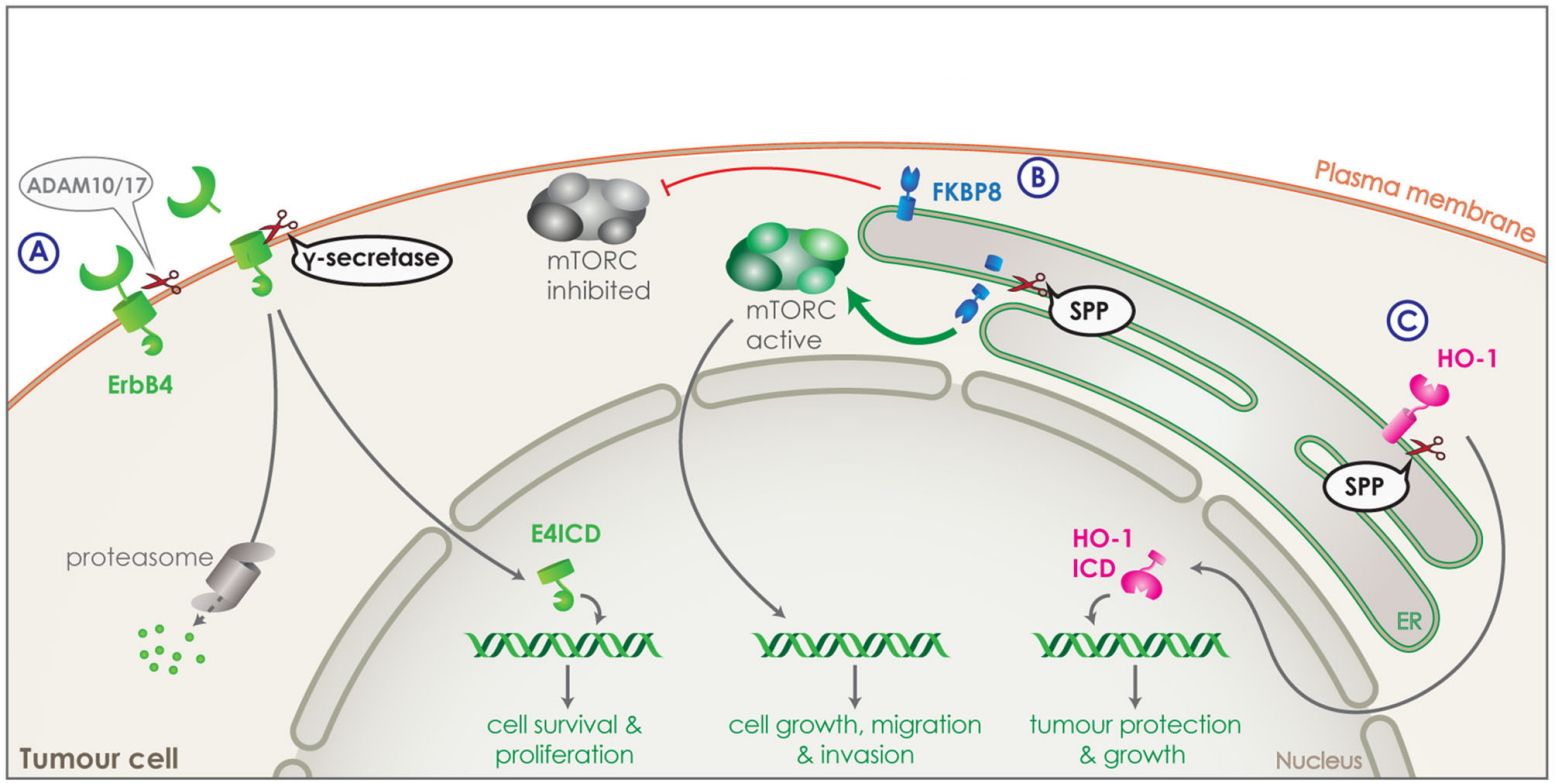
Intramembrane proteolysis in cancer related signaling. **(A)** Receptor tyrosine kinase, ErbB4 (green) is shedded by ADAM10 or 17 and is then cleaved by γ-secretase releasing ErbB4 ICD (E4ICD). The ICD includes the tyrosine kinase domain and can either get degraded by the proteasome or translocate to the nucleus. Increased nuclear translocation of E4ICD in breast cancer cells boosts transcription of genes related to survival and growth. **(B)** FKBP8 (blue) is a tail anchored type-II transmembrane protein in the ER and a physiological inhibitor of the mTORC pathway. SPP can directly cleave FKBP8 and increase of SPP expression by tumor cells can lead to strong reduction of FKBP8 levels and a strong activation of the mTORC pathway. Through its transcriptional functions, mTORC can increase transcription of genes boosting cell growth, migration and invasion of tumor cells. **(C)** Heme oxygenase-1 (HO-1) (pink) is a tail anchored enzyme located in the ER. HO-1 can be cleaved by SPP and the ICD released in the cytosol can translocate to the nucleus. Increased expression of SPP in tumor cells, results in increased nuclear HO-1 ICD, which boost tumor growth and survival.

Another family of molecules involved in signaling that are tightly connected to cell proliferation, differentiation, apoptosis and, thus, also cancer are the receptor tyrosine kinases (RTKs) (124). Half or more of the known RTKs to date are also substrates of γ-secretase following initial shedding by ADAM10 or 17 (Figure 4A). γ-secretase releases RTK ICDs, which include the tyrosine kinase domain and can translocate to the nucleus or are subject to proteosomal degradation. In the nucleus, the RTK ICDs can interact with transcriptional regulators to affect cell proliferation, survival and differentiation, while translocation to the proteasome results in rapid degradation (Figure 4A). ErbB-4 was the first RTK discovered to be cleaved by γ-secretase via RIP generating the ErbB4 ICD (E4ICD) (125). Nuclear translocation of the E4ICD regulates the transcription of genes related to the pro-apoptotic function of ErbB4, while inhibition of γ-secretase blocks the effect on gene expression (125–127). Many more RTKs have been identified as γ-secretase substrates, including EphA4, EphB2, IGF-1R (128, 129). As altered RTK signaling has been linked to carcinogenesis, with for example increased E4ICD promoting growth of breast cancer cells *in vitro* and *in vivo* in mice, dysregulation of the RIP process could promote tumor progression (129, 130).

The participation of γ-secretase activity in cancer progression is not limited to the already mentioned substrates. Numerous more proteins that are involved in the pathology of cancer progression have been shown to undergo cleavage by γ-secretase, as part of a more complex mechanism. Some notable substrates include CD44 (131), E-cadherin (132), and epithelial cell adhesion molecule (EpCAM) (133). However, despite the anti-tumor effect of γ-secretase inhibitors, their adverse effects due to cleavage of multiple substrates make their use in patient treatment very complicated (116).

In addition to PS, also SPP has been implicated in development and progression of cancer. For instance, its expression levels are found to negatively correlate with overall survival and recurrence free survival in lung and breast cancer (76). Two different substrates of SPP have been identified that could be responsible for this effect: heme oxygenase-1 (HO-1) and FKBP8. HO-1 is an enzyme attached to the ER membrane via a C-terminal tail anchor and is responsible for heme-degradation. It is stress-inducible, cytoprotective and highly expressed in numerous cancers, promoting the tumour's survival, growth and angiogenesis (134, 135). Part of HO-1's cancer boosting activity is unrelated to its enzymatic activity, but attributed to the nuclear translocation of HO-1 ICD (Figure 4C). As HO-1 is cleaved by SPP within its membrane-spanning domain, HO-1 ICD production and nuclear translocation is reduced with SPP knockdown and increased with SPP overexpression. Increased expression of SPP by the tumor cells, would thus increase the cleavage of HO-1, protecting the tumor from cellular stress and boosting its growth (Figure 4C) (22, 23).

FKBP8 is a non-canonical member of the FK-506-binding protein (FKBP) family, a tail anchored type-II transmembrane protein located in the ER and an endogenous inhibitor of the mTORC pathway (Figure 4B) (136). FKBP8 was identified as an SPP substrate via a proteomic approach (76). Cleavage of FKBP8 by SPP leads to an overactivation of the mTORC pathway, which in turn causes an increase in cell growth, migration and invasion of tumor cells. This effect could be rescued by suppression of the mTORC pathway demonstrating the involvement of SPP in the mTORC signaling pathway (Figure 4B) (76).

As levels of SPP have been proven to be upregulated in different forms of cancer and often correlate with poor prognosis for the patient, while inhibition of SPP activity *in vitro* seems to suppress the tumor growth (76, 137), it is of interest to consider inhibition of SPP as a treatment method. Despite ongoing research in the field of SPP/SPPL inhibition, the identification or synthesis of specific inhibitors has been unfruitful so far. 1,3-di-(N carboxybenzoyl-leucyl-L-leucyl) amino acetone [(Z-LL)_2_-ketone] is one of the best inhibitors of SPP, which was also used to isolate and identify the protease (17, 138). Although (Z-LL)_2_-ketone spares the activity of γ-secretase, SPPL2c and SPPL3, it inhibits SPPL2a and SPPL2b dependent cleavage (37, 39, 139, 140) making it an unsuitable inhibitor for treatment. A lot of research has been done in an effort to develop specific SPPL2a inhibitors (141, 142) and some of these inhibitors are also targeting SPP while having decreased affinity to other proteases, such as γ-secretase (143). The research on these inhibitors and their structures could be used as a solid basis to develop specific SPP inhibitors in the future. However, even when a specific SPP inhibitor is discovered, its use would have to be carefully considered and restricted due to the numerous important physiological functions of SPP. These functions include cleavage and removal of signal peptides (144), participation in endoplasmic reticulum-associated degradation (ERAD) (145) and others.

## Intramembrane Proteolysis in Signaling of the Immune System

Multiple members of the SPP/SPPL family have been shown to be involved in proper function of the immune system. In most cases, the exact mechanism on how SPP/SPPL proteases affect survival, maturation and function of certain immune cells remains elusive, as is the case with SPPL3's function in natural killer (NK) cells. SPPL3 deficiency results in impairment of NK cell maturation in a cell autonomous manner that is dependent on the catalytic function of SPPL3 (146). In NK-cell specific conditional knockout mice, not only the number of peripheral NK cells was reduced, but also the remaining cells had reduced cytotoxicity and expression of numerous NK cell surface receptors (19, 146). Although, it has been shown that the proteolytic activity of SPPL3 is necessary for the correct maturation of NK cells suggesting the existence of one or more specific substrates involved in this process, these substrates have not been identified so far (146). As NK cells play an important role in natural cancer immunosurveillance, SPPL3 malfunction could be connected to decreased NK cell numbers and thus decreased immunosurveillance (147).

In addition, SPP has also been demonstrated to affect the function of NK cells, but in this case an indirect mechanism was identified. SPP affects the presentation of histocompatibility antigens on the cell surface of healthy cells. Lack of such antigens makes cells a target for NK cells, as they are considered unhealthy. SPP generates these peptides that then bind to HLA-E receptors and are presented on the cell surface, in different ways. Either by cleaving the signal peptide of polymorphic major histocompatibility (MHC) class-I molecules in the ER in collaboration with signal peptidase (148), or from non-MHC-signal peptides (149), or independently of signal peptidase, directly from multipass TM proteins (150). SPP, thus, contributes to multiple mechanisms for providing signals for the circulating NK cells. As NK cells rely on these signals to recognize the organism's cell as healthy, lack of such peptides on the cell surface would cause the circulating NK cells to attack and kill these cells (151, 152). Thus, lack of SPP activity could cause the immune system to attack cells that are otherwise healthy due to lack of “self-recognition” peptides.

**Figure 5:**
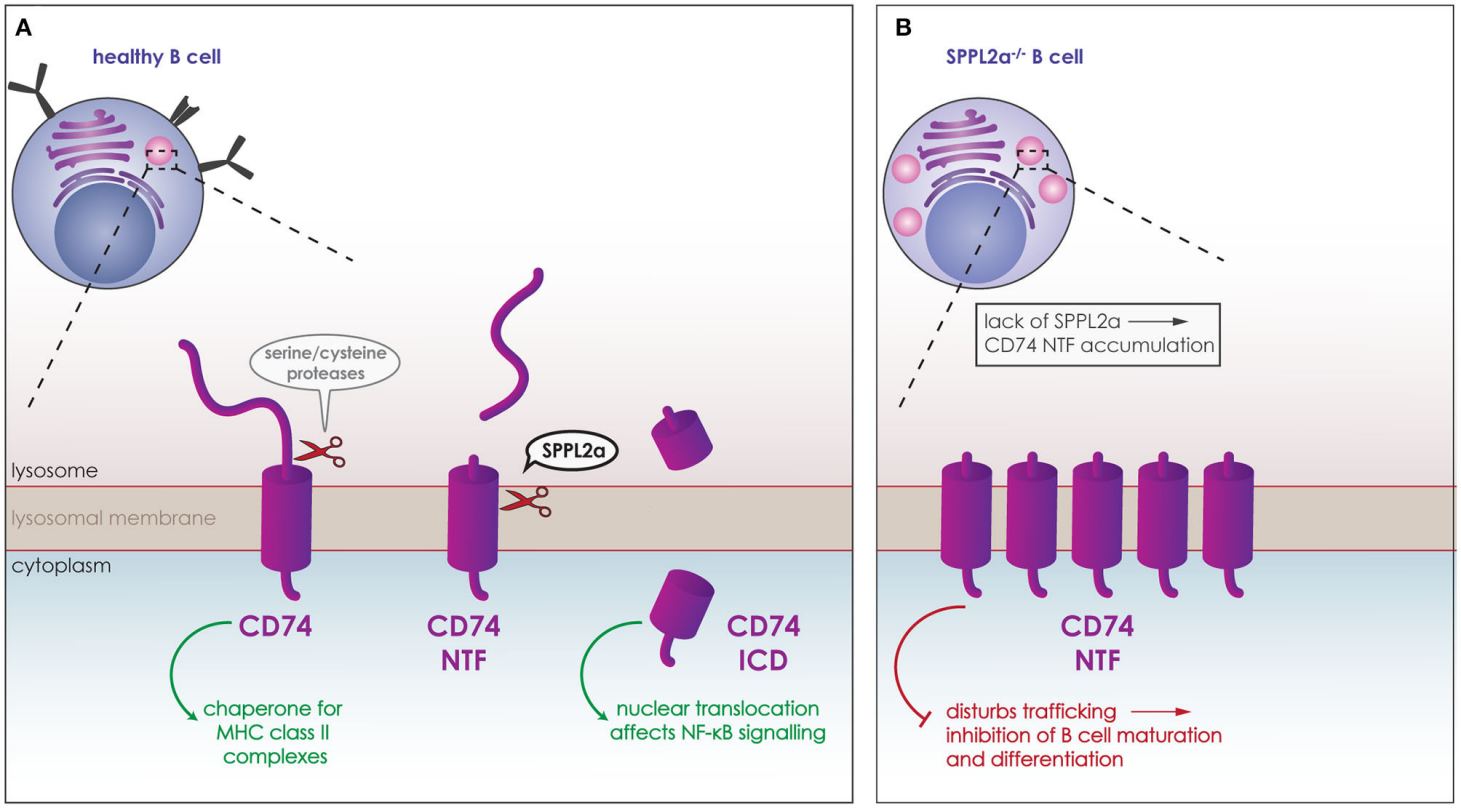
Role of SPPL2a in the maturation of B cells. **(A)** CD74 (purple) is a type-II TM protein expressed in B cells and other antigen presenting cells. It acts as a chaperone for the MHC class II complexes, and is degraded in lysosomes (pink) for antigen-derived peptides to bind to MHC class II. CD74 is cleaved first by serine or cysteine proteases, which release the ectodomain and generate membrane bound CD74 NTF. CD74 NTF is then processed by SPPL2a releasing the CD74 ICD which can translocate to the nucleus and affect NF-κB signaling. **(B)** Lack of SPPL2a (SPPL2a^−/−^) causes an accumulation of CD74 NTF, which disrupts proper trafficking within the cell and blocks B cell receptors from reaching the cell surface resulting in inhibition of B cell differentiation and maturation.

SPPL2a has been found to play a crucial role within the immune system. Compromise of the SPPL2a proteolytic activity causes a defect in the maturation of splenic B cells and conventional dendritic cells (cDCs) (139, 153–155). The substrate of SPPL2a responsible for this phenotype is CD74, the first verified SPPL2a substrate *in vivo* (139, 153, 154). CD74 is a type-II TM protein expressed in antigen presenting cells. It acts as a chaperone for the MHC class II complexes, assuring that no premature peptides bind to the complex. In order for antigen-derived peptides to bind to MHC class II, CD74 is degraded. To this end, it undergoes RIP being cleaved first by serine or cysteine proteases, such as cathepsin S, producing the membrane bound CD74 NTF. This NTF is then cleaved in the transmembrane domain by SPPL2a (Figure 5A) (6, 81). Although the CD74 ICD generated from this proteolytic cleavage can translocate to the nucleus and potentially affect the NF-κB signaling pathway (Figure 5A) (156), lack of CD74 ICD is not responsible for the observed phenotype. Instead, the phenotype is caused by the accumulation of CD74 NTF upon SPPL2a deficiency, which in turn causes structural changes in endocytic compartments and disturbances of trafficking (Figure 5B) (139, 154). As a result of this, presence of the key B cell maturation receptors BAFF and B cell antigen receptor (BCR) on the cell surface is reduced (Figure 5B). Reduced signaling from these receptors would account for the reduced differentiation and maturation (139, 154). Removing expression of both SPPL2a and CD74, rescues the structural changes in the subcellular compartments and the maturation of B cells in mice. This phenotype is also partly conserved in humans as mutations that reduce SPPL2a expression cause an accumulation of CD74 NTF in B cells. Although no maturation defect was observed in humans, the partial loss of cDCs seen in mice was observed in human samples and is thus conserved in both species (155, 157).

Additional known SPPL2a and/or SPPL2b substrates are also involved in proper function of the immune system, such as TNFα and Fas ligand (FasL), however cleavage of these substrates has only been validated in cell culture model systems so far (37–39). Fas ligand (FasL) is a type-II transmembrane protein with very restricted expression that belongs to the superfamily of TNF cytokines. Its binding to the Fas receptor induces apoptotic cell death, and mutations on either the ligand or the receptor can cause autoimmune lymphoproliferative syndrome (ALPS) (158). FasL can undergo RIP, being cleaved initially by ADAM10 and then SPPL2a, which releases the FasL ICD. This ICD can translocate to the nucleus where it performs reverse signaling, reducing the activation-induced proliferation in B and T cells (39, 159). Even though the physiological relevance of the release of the FasL ICD by SPPL2a remains unclear, the reverse signaling performed by the ICD could be important in preventing hyperactivation of the immune system and/or terminating an immune response following antigen stimulation (159). In this case decreased SPPL2a proteolytic activity would prolong immune responses possibly also leading to autoimmunity or allergies, while increased cleavage by SPPL2a could stop the immune response before the threat is under control, showing the importance of fine-tuning such processes.

TNFα is a type II transmembrane protein that undergoes RIP being cleaved first by ADAM17, releasing soluble TNFα, and then by SPPL2a/b releasing TNFα ICD (37, 38). Soluble TNFα is a proinflammatory cytokine, the release of which can be induced in macrophage cell systems by stimulating the cells with lipopolysaccharide (LPS). It has been reported that TNFα ICD, released by SPPL2a/b, can stimulate the expression of Interleukin 12 (IL-12), another proinflammatory cytokine. IL-12, is released by LPS-stimulated dendritic cells, but inhibition or downregulation of SPPL2a and/or SPPL2b, led to an inhibition of IL-12 production by stimulated mature dendritic cells (mDCs). Additional treatment with TNFα ICD rescued IL-12 expression upon stimulation (38). However, it remains unclear whether such a mechanism can be seen *in vivo*.

## Impact of Intramembrane Proteolysis on Subcellular Trafficking

**Figure 6:**
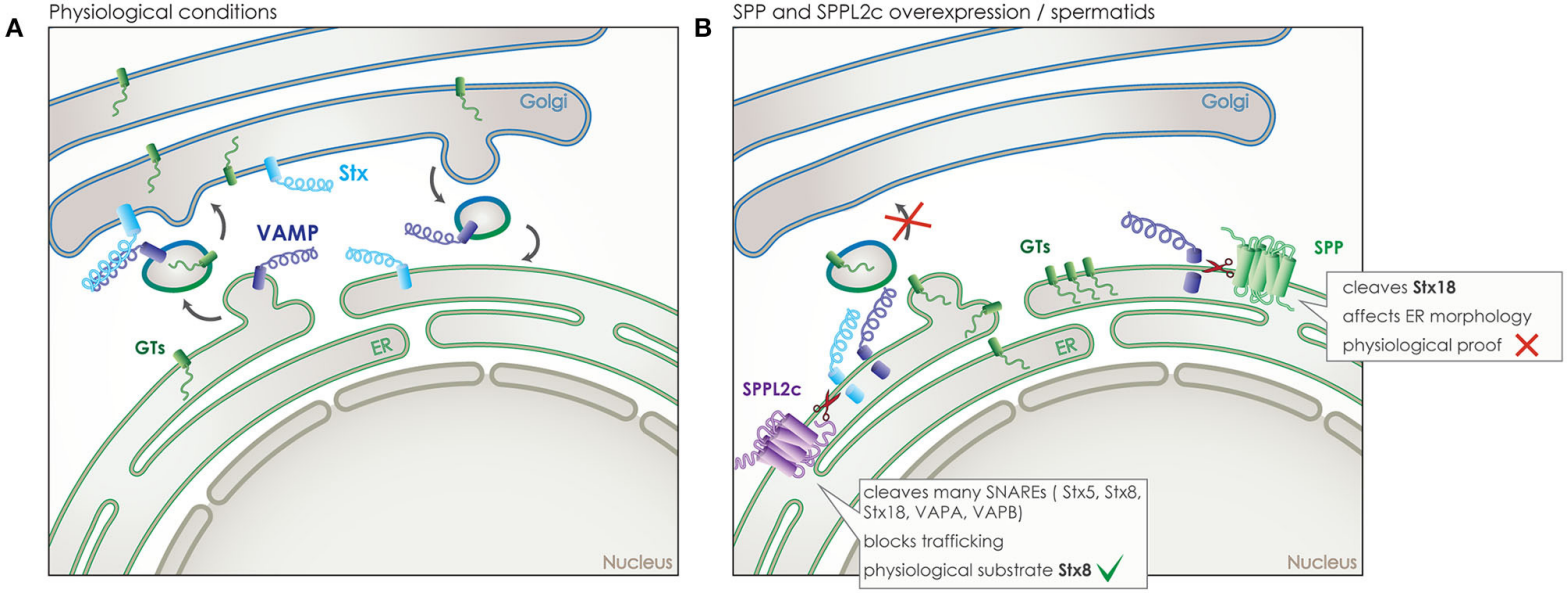
Role of SPP and SPPL2c in membrane trafficking. **(A)** Under physiological conditions, vesicular transport is responsible for transport of proteins between the different membrane organelles of the cell, such as the transport of glycosyltransferases (GTs) (green) from the ER to the Golgi. SNARE proteins assure the specificity of trafficking by controlling membrane fusion. One type of SNAREs, the vesicle-associated membrane proteins (VAMPs) (dark blue), accompany the vesicle, while another type, the syntaxins (Stx) (light blue), is located at the target membrane and awaits the vesicle. Only the correct combination of these two SNAREs enables the fusion of the vesicle to the target membrane. **(B)** SPP overexpressed *in vitro* or SPPL2c expressed physiologically *in vivo* in spermatids, can cleave some of those SNARE proteins, leading to a blockade of the transport between ER and Golgi, an accumulation of the vesicle cargo in the ER and even structural changes of cellular compartments.

Trafficking clearly holds a key role in signaling, as the signaling molecules and receptors need to be present in the correct time and place, both for a signal to be sent but also to be received and processed. SPP/SPPLs do not only affect trafficking indirectly, like in case of CD74, but also directly by cleaving proteins that are central mediators of trafficking. This has been shown for SPP and for SPPL2c, both of which localize to the ER but with SPPL2c being expressed only in a very specific cell type in testis. SPP and SPPL2c are capable of cleaving SNAREs, which are required for targeted and successful fusion of membrane vesicles at their destination and in their majority are tail-anchored type-II (type IV) transmembrane proteins (Figure 6A) (24, 25, 80).

As cleavage of SNARE proteins would cause an inhibitory effect on vesicular trafficking, the wide expression of SPP across species and cell types predisposes for a limited number of substrates in the SNARE family (18). Indeed, so far only one member of the SNARE family, syntaxin 18 (Stx18), has been validated as an SPP substrate (Figure 6B). Nevertheless, given the crucial role of Stx18 in the organization of the ER membranes, ectopic expression of SPP led to a reorganization of ER morphology that could be rescued by co-expression of Stx18 (80). However, the physiological relevance of this phenotype under endogenous conditions remains unclear (19).

Contrary to SPP, SPPL2c expression is extremely restricted. After remaining elusive for many years, expression was finally reported just last year, in testis of male mice and humans, specifically in elongated spermatids, where it resides either in the ER or pre-Golgi compartments (24). The high levels of SPPL2c expression found in this limited number of cells that undergo significant reorganization to form mature sperm, induces compartment reorganization by impairing vesicular trafficking. Ectopically expressed SPPL2c in HEK 293 cells has been shown to cleave numerous SNARE proteins, including Stx5, Stx8, Stx18, VAPA, VAPB, and others, while Stx8 was also identified as an SPPL2c substrate *in vivo* in mouse testis (25). Cleavage of SNARE proteins in HEK293 leads to reduced vesicle transport from the ER, reduced maturation and glycosylation of various secretory and membrane proteins (Figure 6B). Prolonged expression of SPPL2c causes structural alterations on the compartments of the secretory pathway, in particular the ER and the Golgi (25). The pronounced effects of SPPL2c expression on the secretory pathway most likely explain its very restrictive endogenous expression. SPPL2c deficiency in mice leads to reduced mobility of sperm, altered glycosylation pattern of the glycocalyx and apparent defects in the formation of the acrosome (24, 25). As SPPL2c expression was observed in the very same cell type in human samples (24), it is very plausible that it could be involved in male infertility and would be worth investigating. Furthermore, based on the results from mating SPPL2c deficient mice, it appears that SPPL2c would also play a role in the fertility of female mice (24). So far, the limited amount of mature oocytes produced in every cycle and their short life span has not allowed for further research into this aspect, it does remain, however, of high interest as it might be connected to infertility problems in humans.

## Impact of Intramembrane Proteolysis on Calcium Signaling

Calcium (Ca^2+^) acts as a very important second messenger, which is induced by various intracellular signaling cascades. Changes in the intracellular Ca^2+^ concentrations are involved in a variety of cellular processes in both physiological and pathological conditions (160). PS, SPPL3 and SPPL2c have all been associated to calcium-dependent signaling (24, 161). For SPPL3 this effect has been linked to the modulation of NFAT transcription factor activity, but was shown to be independent of its proteolytic function. This is the only non-proteolytic function attributed not only to SPPL3, but to any of the members of the SPP/SPPL family. SPPL3 presence reportedly enhances the interaction between stromal interaction molecule 1 (STIM1) and Orai, thus enhancing the store-operated Ca^2+^ entry (SOCE) leading to Ca^2+^ influx. The interaction of these two proteins and subsequent Ca^2+^ influx play a key role in the transmission of signaling from the T-cell receptor (TCR) to the NFAT transcription factors (161). This effect though it boosts the response to TCR signaling, has so far only been confirmed in cell culture models and its physiological relevance remains unclear.

PSs, as part of the γ-secretase complex, have also been implicated in the regulation of intracellular Ca^2+^ levels and Ca^2+^ signaling (162–164). Although this mechanism appears independent of the proteolytic function of γ-secretase, it is affected by PS mutations that are linked to familial Alzheimer's disease (FAD) (165, 166). PSs are suggested to be involved in this phenotype by a multitude of mechanisms. PSs can form ER Ca^2+^ leaking channels, the function of which is disturbed by the FAD mutations (162). They can also interact with the sarco ER Ca^2+^-ATPase (SERCA) pumps (163) and lastly, release Ca^2+^ from stores through the inositol trisphosphate receptor (InsP_3_R) by agonistic activation (164). The participation of PS in physiological Ca^2+^ signaling and the disturbance of the balance by the presence of FAD mutations is considered as a possible contributor to Alzheimer's disease pathology but remains under debate in the field.

**Figure 7:**
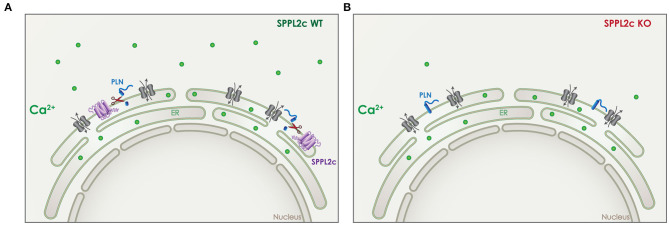
Role of SPPL2c in Ca^2+^ signaling. **(A)** In wildtype (WT) mice, SPPL2c (purple) is expressed in the ER of elongated spermatids, where it cleaves phospholamban (PLN) (blue), a tail anchored type II-transmembrane protein. PLN is involved in the regulation of Ca^2+^ (green) transport from the cytosol to the ER and *vice versa*. **(B)** Lack of SPPL2c in knockout (KO) mice, leads to reduced cleavage of PLN, which disturbs the intracellular Ca^2+^ balance. Reduced levels of cytosolic Ca^2+^ were detected in elongated spermatids lacking SPPL2c expression.

In case of SPPL2c, the effect on calcium-signaling is dependent on cleavage of its *in vivo* validated substrate phospholamban (PLN) (24). PLN is a small tail anchored protein that resides in the ER and can regulate the transport of Ca^2+^ in and out of the ER (Figure 7). In wildtype elongated spermatids, the physiological expression of SPPL2c results in cleavage of PLN and a particular Ca^2+^ balance in those cells (Figure 7A) (24). In mice that lack SPPL2c, PLN levels were increased and cytosolic Ca^2+^ levels were found to be decreased (Figure 7B). This finding might be related to the movement capabilities of mature spermatids (24).

## Impact of Intramembrane Proteolysis on Protein Glycosylation

Glycans are attached to a large number of cellular proteins, mainly transmembrane and secreted proteins, in a variety of combinations and positions. In many cases this can affect the function and localization of these proteins, including functions like cell-cell recognition and communication (167). The precise pattern of glycosylation needs to be tightly regulated as altered glycosylation patterns have been linked to numerous pathologies (168), including cancer (169, 170) and Alzheimer's disease (171). SPPL3 and SPPL2c have both been shown to affect protein glycosylation, in a direct and indirect manner, respectively (25, 28).

**Figure 8:**
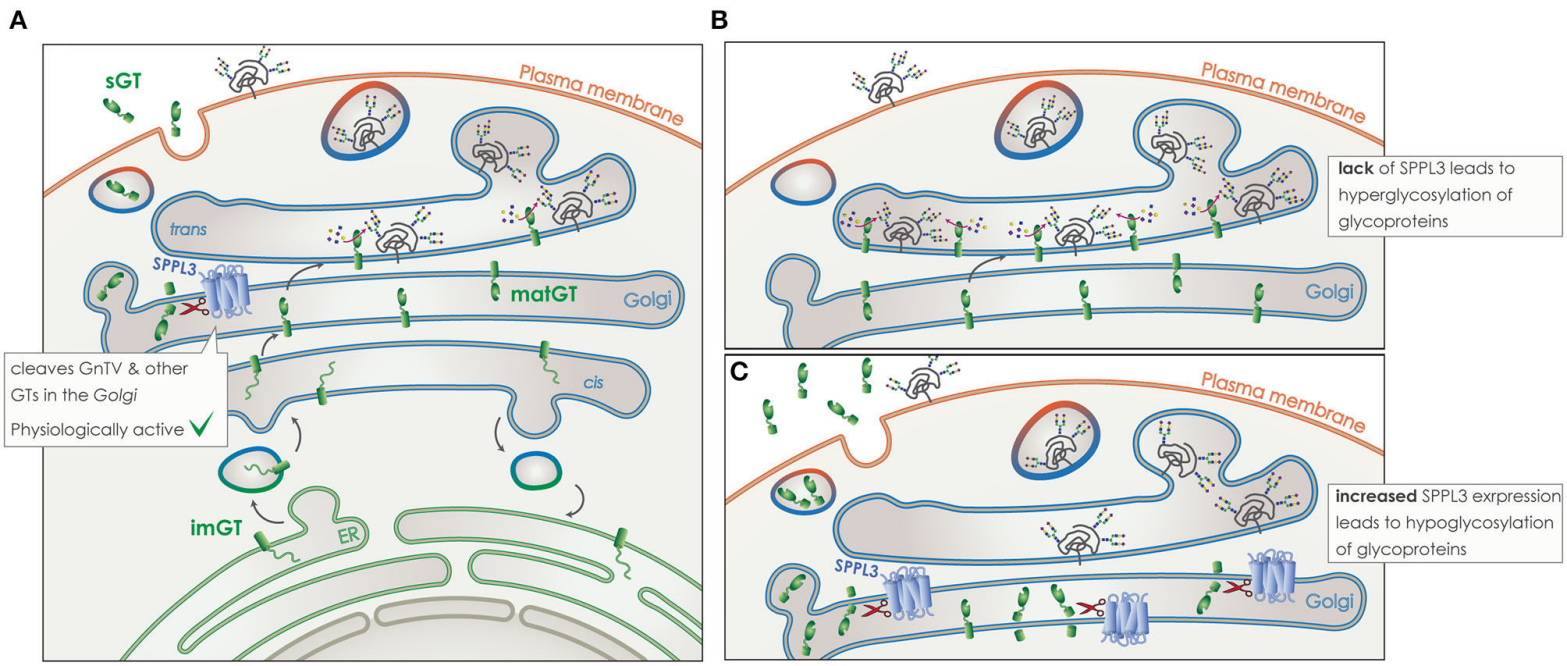
Role of SPPL3 in glycosylation. **(A)** Glycosyltransferases and glycosidases (GT) (green), are transported from the ER to the Golgi in their immature form (imGT). On their passage through the Golgi stacks they mature (matGT). In their mature form they can either add complex N-glycans to glycoproteins (gray), or get cleaved by SPPL3 (blue), resulting in the release of a soluble GT (sGT) that contains the catalytic site. Glycoproteins are then transported to the plasma membrane and their function or time-of-stay at the surface is affected by levels of glycosylation. SPPL3 can affect the balance of this glycosylation. **(B)** Reduced levels of SPPL3, lead to an accumulation of matGTs in the Golgi that results in hyperglycosylation of glycoproteins. **(C)** Increased levels of SPPL3, lead to increased secretion of sGTs and reduced mature enzymes in the Gogli, thus hypoglycosylation of glycoproteins is observed.

SPPL3 directly cleaves numerous type-II TM glycosidases and glycosyltransferases in the Golgi (Figure 8). Cleavage takes place within or in close proximity to the TM domain of the protein and leads to the release of a soluble domain, which can be found in the supernatant of cell culture models, but also in human body fluids like blood (Figure 8A) (172–175). Of note, the secreted ectodomain of the glycan modifying enzymes contains the active site responsible for protein-glycosylation. Although these soluble glycosylating enzymes remain in principle active, the lack of nucleotide activated sugar donors in the extracellular compartment renders them most likely inactive (168, 176, 177). Thus, by controlling the amount of numerous active glycosylating enzymes in the Golgi, SPPL3 can affect the global glycosylation pattern of a cell and consists an easy switch to alter this pattern by affecting a single protein. It has been shown in cell culture and *in vivo* that lack of SPPL3 activity leads to an accumulation and/or decreased secretion of glycan modifying enzymes and, consequently, to hyperglycosylation of a variety of cellular glycoproteins (Figure 8B). While increased activity of SPPL3 has the opposite effect and results both in increased secretion of the soluble glycosidases and hyperglycosylation of glycoproteins (Figure 8C) (28, 29).

The most well-characterized substrate of SPPL3 is N-acetylglucosaminyltransferase V (GnTV), a Golgi localized enzyme that belongs to the family of N-acetylglucosaminyltransferases (GnTs) that are responsible for N-acetylglucosamine (GlcNAc) branching during the formation of complex N-glycans (Figure 8) (28, 178). Interestingly, levels of GnTV have also been found to increase in early stages of many cancers (179, 180). Often the increase of GnTV negatively correlates with patient survival, this is however seen only in specific types of cancer, such as renal cancer (181). Increased presence and activity of GnTV leads to hyperglycosylation of specific cell surface receptors. This increases their presence on the plasma membrane and is connected to cancer growth and metastasis through enhanced growth factor signaling (179). Recent studies are trying to target GnTV in an effort to treat cancer (182). Presence of SPPL3 can strongly affect levels of endogenous GnTV with MEF cells from SPPL3 knockout mice clearly accumulating mature GnTV (28). Reduction of SPPL3 activity would thus, not only interfere with NK cell maturation, but also negatively affect the cellular glycosylation pattern setting up a favorable environment for tumorigenicity. SPPL3 could indeed be involved in tumorigenicity as some single nucleotide polymorphisms (SNPs) in the coding region of SPPL3 have recently been linked to breast cancer as a risk factor (183).

SPPL2c can also affect the glycosylation pattern of glycoproteins, though through a different pathway than SPPL3 as it has been shown that these two proteases do not have substrate overlap (25). Nonetheless, through the cleavage of SNARE molecules SPPL2c disrupts the trafficking of glycosidases and glycosyltransferases (Figure 6). As these proteins need to reach the *trans*-Golgi compartment in order to fully mature and interact with their substrates, numerous glycoproteins with complex N-glycans are hypoglycosylated and do not mature properly upon ectopic expression of SPPL2c (25). This indirect effect of SPPL2c expression on protein glycosylation most likely is also involved in the maturation process of spermatids (25). Under physiological conditions, SPPL2c contributes to the complex and specific glycosylation pattern of the glycocalyx that surrounds mature sperm and is necessary for species specificity in mating, as it ensures the fusion of the ovum with the sperm (184). A lectin microarray analysis of mature sperm showed a difference in the sperm glycan fingerprint when SPPL2c was knocked-out (25). As SPPL2c is physiologically expressed in humans it would be of interest to check if any variations in the proteases expression and/or function can be connected to infertility. Interestingly, the *SPPL2C* gene is found to be affected in patients suffering from the Koolen-de Vries 17q21 microdeletion syndrome (185, 186). Six genes are in total affected by this deletion that results mainly in mental retardation, however one of the male patients was also found to be infertile (187). The restricted expression of SPPL2c in the organism makes it very unlikely for the protease to contribute to the mental phenotype, however it remains highly probable that it contributes to the fertility phenotype. Further research on the topic and analysis of samples from individuals suffering from infertility could be of great interest.

## Conclusion

In the past 20 years, a lot of progress has been made in the field of intramembrane proteolysis in general and in context of GxGD aspartyl proteases in particular. With the identification of multiple substrates for these proteases, we now better understand their connection to multiple pathways including the vascular system, cancer progression and many more. We can also appreciate the complexity of their function and the difficulties in targeting specific protease-substrate combinations. However, in addition to the research performed this far, in order to best understand and target GxGD aspartyl proteases, we would need not only specific inhibitors, like the ones developed by Novartis for SPPL2a (141–143), but also an even more comprehensive understanding of their physiological activity. The lethal phenotype demonstrated by mice lacking some of these proteases in the early developmental stages had posed an obstacle in the past years, nonetheless, the generation of conditional knockout mouse lines will enable leaps of progress in this field.

## Author Contributions

AP and RF wrote the manuscript. Both authors contributed to the article and approved the submitted version.

## Conflict of Interest

The authors declare that the research was conducted in the absence of any commercial or financial relationships that could be construed as a potential conflict of interest.
